# Evaluation of Early Warning, Alert and Response System for Ebola Virus Disease, Democratic Republic of the Congo, 2018–2020

**DOI:** 10.3201/eid2712.210290

**Published:** 2021-12

**Authors:** Mory Keita, Héloïse Lucaccioni, Michel Kalongo Ilumbulumbu, Jonathan Polonsky, Justus Nsio-Mbeta, Gaston Tshapenda Panda, Pierre Celeste Adikey, John Kombe Ngwama, Michel Kasereka Tosalisana, Boubacar Diallo, Lorenzo Subissi, Adama Dakissaga, Iris Finci, Maria Moitinho de Almeida, Debarati Guha-Sapir, Ambrose Talisuna, Alexandre Delamou, Stephanie Dagron, Olivia Keiser, Steve Ahuka-Mundeke

**Affiliations:** Institute of Global Health, University of Geneva, Geneva, Switzerland (M. Keita, J. Polonsky, S. Dagron, O. Keiser);; World Health Organization Regional Office for Africa, Brazzaville, Congo (M. Keita, A. Talisuna);; European Centre for Disease Prevention and Control, Stockholm, Sweden (H. Lucaccioni, I. Finci);; Ministère Provincial de la Santé, Goma, Democratic Republic of the Congo (M.K. Ilumbulumbu, M.K. Tosalisana);; World Health Organization, Geneva (J. Polonsky, L. Subissi);; Ministère de la Santé, Direction Générale de la Lutte contre la Maladie, Kinshasa, Democratic Republic of the Congo (J. Nsio-Mbeta, G.T. Panda, P.C. Adikey, J.K. Ngwama);; Ministère de la Santé, Direction Régionale de la Sante du Plateau central, Ziniaré, Burkina Faso (A. Dakissaga);; Centre for Research on Epidemiology of Disasters, Research Institute on Health and Society, Université Catholique de Louvain, Brussels, Belgium (M.M. de Almeida, D. Guha-Sapir);; Africa Center of Excellence (CEA-PCMT), University Gamal Abdel Nasser, Conakry, Guinea (A. Delamou);; Institut National de Recherche Biomédicale, Kinshasa (S. Ahuka-Mundeke)

**Keywords:** Ebola, Ebola virus infection, viruses, Early Warning Alert and Response System, EWARS, surveillance, Democratic Republic of the Congo, DRC, outbreak

## Abstract

The 10th and largest Ebola virus disease epidemic in the Democratic Republic of the Congo (DRC) was declared in North Kivu Province in August 2018 and ended in June 2020. We describe and evaluate an Early Warning, Alert and Response System (EWARS) implemented in the Beni health zone of DRC during August 5, 2018–June 30, 2020. During this period, 194,768 alerts were received, of which 30,728 (15.8%) were validated as suspected cases. From these, 801 confirmed and 3 probable cases were detected. EWARS showed an overall good performance: sensitivity and specificity >80%, nearly all (97%) of alerts investigated within 2 hours of notification, and good demographic representativeness. The average cost of the system was US $438/case detected and US $1.8/alert received. The system was stable, despite occasional disruptions caused by political insecurity. Our results demonstrate that EWARS was a cost-effective component of the Ebola surveillance strategy in this setting.

Early case detection is important to control and prevent infectious disease outbreaks (*1*). The 5 identified purposes for early detection surveillance are detecting the first case of the disease in a population previously free, detecting new cases in an area already infected, early detection of an abnormal increase in the level of a disease normally present at a base level, screening for individual cases of noncommunicable diseases, and the first detection of an invasive species in an area previously free of that species (*2*). The International Health Regulations (2005) (*3*) impose obligation on countries to develop, strengthen, and maintain their capacities to detect, verify, assess, report, and respond to any events that may constitute a public health risk and thereby prevent international spread. Public health surveillance systems are poorly developed in many low-income and middle-income countries, as demonstrated by recent Ebola outbreaks, which had devastating consequences in the health and economy of several countries (*4*–*7*).

Ebola virus disease (EVD), if not detected and reported early, can rapidly spread and result in high rates of illness and death (*8*,*9*). In recent years, the world has faced the 2 largest EVD epidemics in recorded history, both of which were declared public health emergencies of international concern by the director-general of the World Health Organization (WHO).

EVD case definitions are crucial surveillance tools, both for referring suspected cases and as screening tools to aid admission and laboratory testing decisions at health facilities (*10*). WHO has developed standard case definitions for alert, suspected, probable, and confirmed cases in the context of routine and community-based surveillance (*11*,*12*) (Appendix Table).

Insufficient command of these case definitions at the community and health-facility level has resulted in late detection of EVD outbreaks. For instance, recent epidemics in both West Africa and the Democratic Republic of the Congo (DRC) were officially declared 3 months after the effective start of the epidemics (*13*,*14*). The epidemic in DRC was the second largest EVD outbreak ever documented after the West Africa EVD epidemic (2013–2016); a total of 3,481 cases (3,323 confirmed and 158 probable) and 2,299 deaths were recorded in August 2018–June 2020 in North Kivu, Ituri, and South Kivu Provinces. This outbreak was particularly complex because it occurred in an active conflict zone (*15*). Public health performance indicators at the beginning of this EVD response were poor, including many community deaths, poor contact tracing, and delays between symptom onset and case isolation. A decline in incidence toward the end of 2019 was thought to be the result of improvement in the quality of surveillance activities, including prompt investigation, early detection and isolation of cases, enhanced community-based surveillance, rapid follow-up of high-risk contacts, and an adaptive vaccination strategy (*16*).

Soon after the declaration of the 10th EVD outbreak in the DRC, an Early Warning, Alert and Response System (EWARS) was implemented throughout North Kivu and Ituri Provinces, to report, collect, investigate, validate, and take early action (isolation, safe burial, or referral) on alerts that met the suspected case definition for EVD. We describe and evaluate this system as implemented in the subcoordination of Beni, established to manage the response across several health zones.

## Methods

### Description of the EWARS

**Figure 1:**
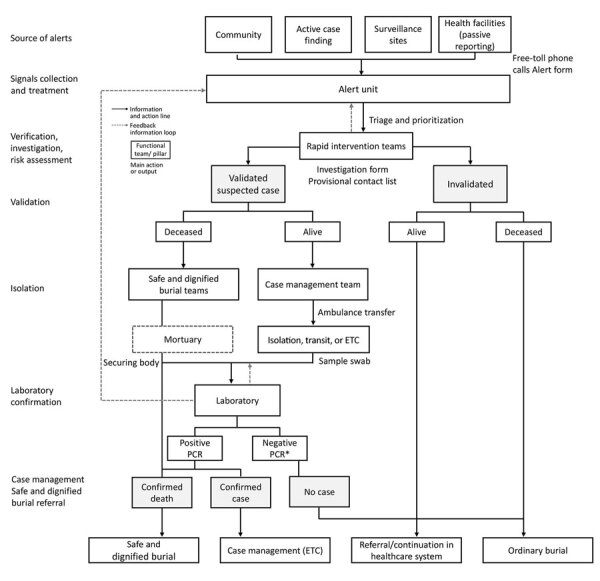
Organization of the Early Warning, Alert and Response System as used in the Democratic Republic of the Congo, August 2018–June 2020. Asterisk (*) indicates 1 negative result for a deceased suspected case-patient or 2 negative results within 72 hours for an alive suspected case-patient. ETC, Ebola treatment center.

The Alert Unit was the core functional unit around which the EWARS was organized (Figure 1); it was composed of an overall operational leader who coordinated activities, a database and information administrator, a case management leader, a Safe and Dignified Burial (SDB) leader, 3 telephone operators, 1 alert monitoring officer, 1 database manager, 1 data clerk, and 1 archivist. The main role of the Alert Unit was to gather and scan alerts from various sources, coordinate the field investigations with the rapid intervention teams, and, if relevant, organize the referral and ambulance transfer or safe burial in collaboration with the case management or SDB team. All alerts and their outcomes were entered and archived into paper-based alert and investigation forms and a Microsoft Excel database (https://www.microsoft.com). There were 4 main sources of alerts: community, in which community health workers, community members, and political and administrative authorities raised alerts; active case finding conducted in health facilities and other structures (pharmacies, churches, traditional practitioners); surveillance sites, including contact tracing teams, vaccination sites, and points of entry/points of control (PoE/PoC); and finally, public and private health facilities that ensured passive reporting.

Two toll-free numbers were activated on August 26, 2018, to enable rapid and easy alert reporting from all the sources. Calls were directed to telephone operators in the Alert Unit, which was operational 24 hours per day, 7 days per week; a smaller team for night shifts comprised the operations leader and phone managers only. In active case finding, passive reporting, or PoE/PoC, the alert notifier completed an alert form, or for community and contact tracing alerts, the telephone operator or alert monitoring officer completed the form. When telephone operators received the alerts, they checked for duplication and conducted preliminary triage to prioritize them by epidemiologic and clinical factors. Rapid intervention teams were then notified to investigate the alert onsite.

Rapid intervention teams were made up of a field epidemiologist, an infection prevention and control (IPC) officer, a communication officer, and a psychosocial worker. All these response pillars were positioned in each health area covered by the alert system, from which a senior epidemiologist would organize rapid intervention teams. The investigation consisted of a detailed history, assessment of the epidemiologic link, clinical symptoms for validation against the suspected case definition (*17*), and initial listing of contacts. Investigation forms were stored in the Alert Unit, and copies were sent to Ebola treatment centers (ETC) for patients requiring admission. The rapid intervention team validated or invalidated the alert on the basis of the investigation findings and provided immediate feedback to the Alert Unit contact persons, who coordinated the next steps. 

For invalidated alerts, the family can proceed with ordinary burial of deceased patients, whereas living patients were referred to public healthcare facilities for free healthcare. Living patients with validated alerts were immediately transferred to a transit center, isolation center, or ETC, depending on the patient’s condition and location. There was no additional validation at triage in ETC. To reduce the risk that a transfer would refuse a patient, the intervention team would propose 2 options according to patient condition and preference: transfer the patient by ambulance or by motorcycles driven by Ebola survivors. After admission to the isolation center, patients followed the suspected case management algorithm: blood samples were taken and tested by using GeneXpert (Cepheid, https://www.cepheid.com) within 3 hours after admission. Cases confirmed by PCR were immediately admitted to an ETC for treatment. Those patients with an initial negative test were discharged pending a second negative result 72 hours later.

The SDB team were notified of validated alerts of deceased patients, then joined the rapid intervention team onsite to engage with the family. The body was secured and a swab sample taken and sent to the laboratory for testing. With family consent, SDB proceeded immediately. However, if the family refused, the body was kept at the mortuary until the laboratory result was available. If the result was negative, the body was returned to the family to proceed with ordinary burial; if the results was positive, SDB was mandatory and enforced by authorities.

### Evaluation Approach and Data Sources and Indicators

We conducted a quantitative evaluation according to guidelines published by WHO (*18*) and the US Centers for Disease Control and Prevention (CDC) (*19*). We used the anonymized Alert Unit database, covering the health zones of Beni, Mutwanga, and Oicha, during August 5, 2018–June 30, 2020, to assess EWARS using the EVD suspected case definition as the standard. An alert was considered validated if it met the definition of an alert case by community-based surveillance or the definition of a suspected case by mobile teams or health stations or centers (*12*). An investigator would validate a suspected case on the evidence of clinical signs in the patient (Appendix Figure).

To assess the true sensitivity, specificity, positive predictive value (PPV), and negative predictive value (NPV) would require laboratory testing for all patients, which would not have been feasible. We calculated sensitivity as the proportion of alerts validated among all alerts meeting the suspected case definition, specificity as the proportion of invalidated alerts among all alerts not meeting the suspected case definition, PPV as the proportion of alerts that met the suspected case definition among all validated alerts, and NPV as the proportion of alerts that did not meet the suspected case definition among all invalidated alerts. We assessed timeliness as the median, range, and interquartile range (IQR) of the delay between the transmission of alert to the Alert Unit and the start of the onsite investigation. We evaluated representativeness through the geographic and demographic coverage of the alerts by comparing alert incidence by sex, age group, and health zone. We appraised usefulness by considering the number of confirmed and probable cases that were detected through the alert system. Finally, we assessed stability by considering how the system was operating over time, disruptions, and sustainability of functioning beyond the emergency response phase, notably in relation with costs and human resources. We conducted all analyses using R statistical software version 4.0.3 (*20*).

## Results

### Outcomes of EWARS

**Figure 2:**
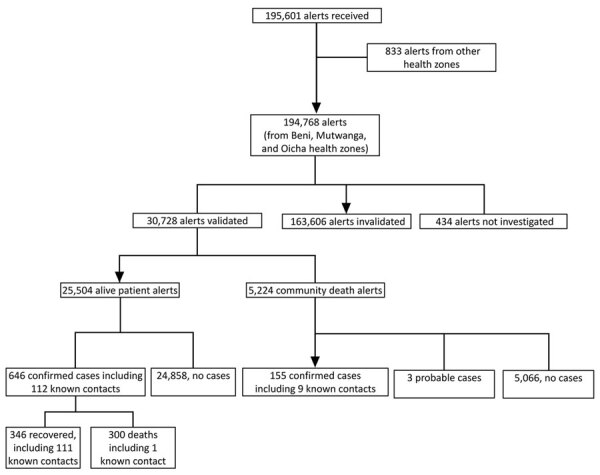
Flow diagram of alerts in the Early Warning, Alert and Response System and their outcomes in 3 health zones, Democratic Republic of the Congo, August 2018–June 2020.

During the study period, 195,601 alerts were received; 194,768 (99.6%) from the health zones of Beni, Mutwanga, and Oicha, and 833 (0.4%) from other health zones (Figure 2). A small number (52,240, 2.7%) were reports of community deaths.

**Figure 3:**
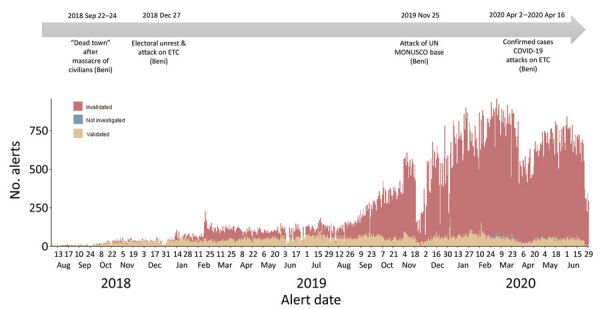
Trend in daily number of alerts from the Early Warning, Alert and Response System by final validation status in 3 health zones, Democratic Republic of the Congo, August 2018–June 2020. Key security incidents during the epidemic period are depicted along the timeline above the graphic. MONUSCO is the name of the UN peacekeeping force in the country. ETC, Ebola treatment center.

On average, there were 280 alerts/day (range 2–955, median 127 alerts/day), although this value greatly varied over time. The number of daily alerts increased progressively, from 6 at the outset in August 2018 to a peak of 922 at the beginning of March 2020. We observed multiple sudden, short-lived decreases in the daily number of alerts, particularly in mid-November 2019 and early April 2020, coinciding with security incidents (see Stability) (Figure 3).

A total of 30,728 (15.8%) alerts were validated as suspected cases. Among those, 801 (2.6%) were finally classified as confirmed cases and 3 (<0.1%) as probable cases. No invalidated alerts became confirmed cases; the information recorded the first time remains in the database, and a new alert with the same information could be quickly detected.

Most (62.6%) alerts were raised by active case finding teams, followed by passive reporting from health facilities (19.0%), and community alerts (15.0%). The remainder (3.6%) originated from other sources (Table 1).

**Table 1:** Characteristics of Ebola virus disease alerts received in Beni subcoordination, Democratic Republic of the Congo, August 5, 2018–June 30, 2020

Characteristic	No. (%) alerts, n = 194,768
Year	
2018	3,211 (1.6)
2019	67,579 (34.7)
2020	123,978 (63.7)
Final alert status	
Invalidated	163,606 (84.0)
Validated	30,728 (15.8)
Not investigated	434 (0.2)
Alert initial status	
Deceased	5,230 (2.7)
Alive	189,538 (97)
Final case classification	
Not a case	193,964 (99.6)
Confirmed case	801 (0.4)
Probable	3 (<0.1)
Source of alert	
Active case finding	121,970 (62.6)
Health structure	36,911 (19.0)
Community	28,928 (15.0)
Other surveillance sites	6,959 (3.6)
Health zone	
Beni	167,503 (86.0)
Mutwanga	12,891 (6.6)
Oicha	14,374 (7.4)
Sex	
F	109,605 (56.3)
M	84,442 (43.4)
Unknown	721 (0.4)
Age group	
0–4	45,934 (23.6)
5–9	22,220 (11.4)
10–19	36,825 (18.9)
20–29	37,945 (19.5)
30–39	21,975 (11.3)
40–49	11,186 (5.7)
50–59	6,668 (3.4)
>60	8,679 (4.5)
Unknown	3,336 (1.7)
Known contact of confirmed or probable case	
No	194,052 (99.6)
Yes	672 (0.3)
Unknown	44 (0.1)

### Sensitivity, Specificity, Positive Predictive Value, Negative Predictive Value

We excluded 434 alerts (0.2%) that were not investigated and 201 alerts (0.1%; 197 invalidated and 4 validated) that could not be classified according to the case definition because of missing data. A total of 17,927 (9.2%) alerts met the EVD suspected case definition. Sensitivity was 84.6% (95% CI 84.1%–85.1%) and specificity 91.2% (95% CI 91.0%–91.3%). PPV was 49.4% (95% CI 48.8%–49.9%) and NPV 98.3% (95% CI 98.2%–98.4%) (Table 2).

**Table 2:** Evaluation results and overall characteristics of Ebola virus disease alerts from EWARS, Democratic Republic of the Congo, August 5, 2018–June 30, 2020*

Alert system	Suspected case definition		Total	% (95% CI)			
	No. met	No. unmet		Sensitivity	Specificity	PPV	NPV
Validated	15,163	15,561	30,724				
Invalidated	2,764	160,645	163,409				
Total	15,245	184,104	194,133	84.6 (84.1–85.1)	91.2 (91.0–91.3)	49.4 (48.8–49.9)	98.3 (98.2–98.4)

*Total excludes 434 (0.2%) alerts that were not investigated and 201 (0.1%) alerts that could not be classified according to the case definition due to missing data. EWARS, Early Warning, Alert and Response System; NPV, negative predictive value; PPV, positive predictive value.

Indicators varied with time, health zone, and source of notification (Table 3). Overall, sensitivity increased over time, and specificity remained high throughout the study period. PPV decreased while NPV increased, which is consistent with the outbreak dynamics and the decrease in incidence toward the end of the epidemic (Appendix Figure).

**Table 3:** Evaluation of EWARS alerts by source of Ebola virus disease alert and health zone, Democratic Republic of the Congo, August 5, 2018–June 30, 2020

Category	% (95% CI)			
	Sensitivity	Specificity	PPV	NPV
Source of alert				
Active case finding/IPC	87.5 (86.9–88.1)	91.7 (91.6–91.9)	51.2 (50.4–51.9)	98.7 (98.6–98.7)
Community	91.4 (90.1–92.7)	93.6 (93.3–93.9)	48.3 (46.6–50.0)	99.4 (99.3–99.5)
Health facility	65.4 (63.8–67.0)	96.2 (96.0- 96.4)	64.5 (62.9–66.1)	96.4 (96.2–96.6)
Other surveillance sites	98.0 (97.4–98.7)	34.3 (33.0–35.6)	33.0 (31.7–34.2)	98.1 (97.5–98.8)
Health zone				
Beni	94.8 (94.4–95.2)	90.6 (90.5–90.8)	44.9 (44.3–45.5)	99.5 (99.5–99.6)
Mutwanga	54.9 (52.4–57.3)	96.4 (96–96.7)	68.2 (65.7–70.8)	93.8 (93.3–94.2)
Oicha	64.3 (62.8–65.8)	93.3 (92.8–93.8)	78.6 (77.2–80.1)	87.2 (86.6–87.9)

*EWARS, Early Warning, Alert and Response System; IPC, Infection Prevention and Control; NPV, negative predictive value; PPV, positive predictive value.

Sensitivity was higher for alerts arising from surveillance sites (98.0%, 95% CI 97.4%–98.7%), community alerts (91.4%, 95% CI 90.1%–92.7%), and active case finding (87.5%, 95% CI 86.9%–88.1%) and lower for those arising from passive reporting from health facilities (65.4%, 95% CI 63.8%–67.0%). Conversely, specificity was highest in health facilities (96.2%, 95% CI 96.0%–96.4%), and was high (>90%) for all other sources except surveillance sites. Sensitivity was higher in Beni (94.8%, 95% CI 94.4%–95.2%) than in Mutwanga (54.9%, 95% CI 52.4%–57.3%) and Oicha (64.3%, 95% CI 62.8%–65.8%), but specificity was higher in Mutwanga (96.4%, 95% CI 96%–96.7%) and Oicha (93.3%, 95% CI 92.8–93.8).

### Timeliness

An investigation was initiated within 2 hours from the time of alert for 188,184 (96.6%) alerts. The median time from alert transmission to the arrival of the investigation team on site was 11 minutes (IQR 10–15 minutes). Information about the time of investigation was not available for 3,475 (1.8%) alerts.

**Figure 4:**
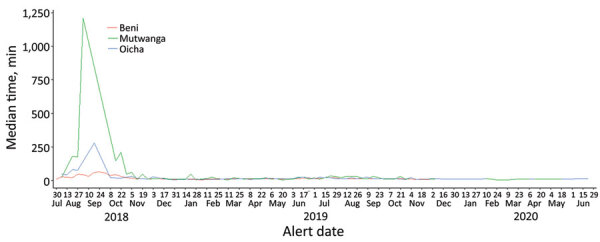
Timeliness over time of alerts from the Early Warning, Alert and Response System, Democratic Republic of the Congo, August 2018–June 2020. Timeliness is defined as weekly median time (in minutes) from alert transmission to the start of the investigation.

Timeliness of responses varied over time with substantial delays were observed at the outset of the system implementation, with greatest delays in Mutwanga (Figure 4). We saw no marked difference in timeliness by source of notification.

### Representativeness

**Figure 5:**
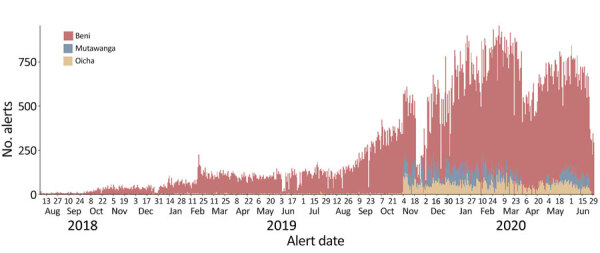
Trend in daily number of alerts in the Early Warning, Alert and Response System in 3 health zones in the Democratic Republic of the Congo, August 2018–June 2020.

We observed substantial variations in the alert incidence between the health zones. On average, there were 241 (range 2–789) alerts/day in Beni, 42.3 (range 1–181) alerts/day in Mutwanga, and 25.4 (range 1–138) alerts/day in Oicha. The alert incidence in the population followed a similar pattern: an average of 36 alerts/1,000 inhabitants/week in Beni, 2.5 alerts/1,000 inhabitants/week in Oicha, and 2.4 alerts/1,000 inhabitants/week in Mutwanga. In Beni, the incidence of alerts increased progressively from the outset (Figure 5). However, in Mutwanga and Oicha, incidence remained low until the beginning of November 2019, when it rapidly increased following community transmission. 

We observed more alerts among female (56.3%) than male (43.4%) patients. Children <5 years of age were the most represented (23.6%), followed by patients 20–29 years of age (18.9%) and 10–19 years of age (18.9%); these percentages approximate the age and sex breakdown of the local population, with the exception of children 5–9 years of age, who were underrepresented (11%).

### Usefulness and Cost

The EWARS system led to the detection of 801 confirmed and 3 probable cases, which equates to 242 alerts notified and 38 alerts validated for each case detected by the system. The total direct and indirect costs associated with EWARS implementation and maintenance was US $353,525 over the 2-year period of operation (Table 4), yielding a minimum value of US $1.8/alert and US $438/case detected.

**Table 4:** Costs for EWARS in Beni, Mutwanga, and Oicha, Democratic Republic of the Congo, August 2018–June 2020*

Health zone	Item	Implementation period costs, USD			Total cost, USD
		2018 Aug 5–Dec 31	2019 Jan 1– Dec 31	2020 Jan 1–Jun 30	
Beni	Prime staff for alerts management teams	6,000	25,200	12,600	43,800
	Prime staff for data managers	900	10,350	5,400	16,650
	Ambulance rental	6,000	72,000	36,000	114,000
	Fuel	9,600	36,000	18,000	63,600
	Purchase of telephones	175	NA	NA	175
	Purchase of materials†	5,500	12,000	6,000	23,500
	Communication credit	750	3,600	2,100	6,450
	Green numbers‡	15,200	15,200	NA	30,400
Oicha	Prime for alerts management teams	NA	12,150	6,300	18,450
	Prime for data managers	NA	5,400	2,700	8,100
	Communication credit	NA	1,200	600	1,800
Mutwanga	Prime for alerts management teams	NA	7,200	5,400	12,600
	Prime for data managers	NA	7,200	5,400	12,600
	Communication credit	NA	800	600	1,400
Total		44,125	208,300	101,100	353,525

*Expenditures included direct and indirect costs. EWARS, Early Warning, Alert and Response System; NA, not applicable. †Flip charts, markers, printed forms. ‡Telephone numbers 0820800001 and 0999009405, which health workers and community members could use for no charge. WHO covered this expense.

### Stability

The alert system operated 24 hours per day, 7 days per week, including a minimal night team to ensure continuity. Continuous communication and reporting of alerts was possible by a comprehensive and stable mobile phone coverage covering all health areas. As such, alerts were collected and analyzed on a continuous basis, and reports were produced and distributed daily. However, despite the continuous availability of human resources and communication networks, the system was severely disrupted by security incidents. Security incidents coincided with decreases in the number of alerts, affecting both the reporting and investigation of alerts (Figure 3). The Alert Unit ceased operations following the standard 90-day period of heightened surveillance after the declaration of the end of the outbreak, as determined by WHO (*21*).

## Discussion

During August 2018–June 2020, EWARS led to the notification and investigation of 194,768 alerts and the detection of 801 confirmed and 3 probable EVD cases. The evaluation showed an overall good performance of the system regarding the main attributes we assessed, highlighting the many strengths of such a system. However, it also revealed disparities in performance between the health zones covered by the system, reflecting differences in the timing of implementation and, most notably, unequal operating conditions (e.g., security incidents).

This system encompassed both event-based and indicator-based surveillance (*22**,**23*), resulting in 7.8% of alerts meeting the definition of EVD suspected case, 4-fold higher than the event-based surveillance system at the community level during the Ebola outbreak in Sierra Leone in 2014–2016, and a 6-fold higher 49.4% PPV (*24*). Approximately 92% of our alerts did not meet the suspected case definition because of a time lag of days between symptom onset, on which the alert launch was based, and the symptoms that were actually present in these patients during investigation.

Although the overall proportion of detected cases among alerts was low (0.4% of all alerts), EWARS aimed to be highly sensitive; actions taken around those confirmed cases successfully interrupted transmission chains and prevented further spread of the disease. Indeed, the system showed a high sensitivity and specificity (>80%) and a low PPV, which reflects the low EVD incidence in the population. All health areas covered by the system reported alerts that did not differ greatly from the population structure, thus suggesting a good demographic representativeness. The system presented prompt timeliness of investigation of alerts throughout its 2 years of operation. Finally, the minimum cost per alert or cases was relatively low compared with that for a nationwide telephone alert system established for rapid notification and response during the 2014–2015 Ebola disease epidemic in Sierra Leone (*25*).

This good performance of EWARS can be explained by the intensive, comprehensive, and continuous reporting flow. First, the system relied on the use of various sources of alerts, involving both passive and active case reporting from the community, health structures, and other surveillance sites. Second, it built upon a stable and extensive telephone network further supported by toll-free numbers, a means of communication that is easily accessible, acceptable, and already commonly used by all stakeholders involved in surveillance. Third, it adopted a decentralized approach for the organization of the investigation teams, which enabled comprehensive coverage of all health areas and prompt reactivity for early action. The existence of a dedicated team at the subcoordination level further supported the coordination of activities at the local level while aiding in the centralization and consolidation of the information circuit. The unceasing availability of all key actors of the reporting system (surveillance, investigation teams, alert unit, and case management/SDB) ensured the continuous reporting and actions around alerts in timely manner. How fast a system detects and responds effectively to a threat is the optimal measure of performance. Continuously evaluating and improving timeliness can identify performance bottlenecks and help to accelerate progress, improving detection speed and response quality (*26*).

The alert system performed better in Beni for all attributes we studied. In Mutwanga and Oicha, sensitivity was <80%, alert incidence was low (even after an increase in the number of daily alerts in late 2019), geographic coverage appeared less comprehensive as many health areas reported few alerts, and delays in investigation were longer, particularly at the outset. Mutwanga and Oicha are 2 rural health zones located at the epicenter of nonstate armed groups’ territories, which greatly affected the operations. Surveillance and investigation activities faced regular security incidents and restrictions, long distances to alert sites, and poor road networks in many health areas. In this context, the alert system was initially implemented in Beni and progressively extended and strengthened in Mutwanga and Oicha. For example, in the early phase, rapid intervention teams were staffed in the Beni subcoordination office only, such that alert investigations in Mutwanga and Oicha suffered longer delays. Surveillance and reporting capacities were also weaker in Mutwanga and Oicha. In November 2019, a training of response personnel (registered nurses, supervisors, and investigators) was organized to address the low incidence of alerts; to strengthen data management capacities, data managers were deployed, leading to a rapid increase in alerts from these health zones.

Despite the effects of security incidents, the EWARS continued to operate throughout the whole period, managing an increasing volume of alerts, leading to the detection of hundreds of cases. In a context of limited surveillance capacities and weak health systems, such an intensive and steadily reporting alert system was vital for the early detection of cases and interruption of the spread of the disease in the population. However, the system was conceived and implemented in an ad hoc manner within the framework of the Ebola outbreak response, which limited its sustainability beyond the resources and time period of the outbreak response. The financial, logistical, and human resources needed to implement and maintain the system were made possible by dedicated response funds and the time-bound engagement of both national support teams and international financial and technical partners. The EWARS ceased operations within 12 weeks of the declared end of the outbreak. The long-term sustainability of systems such as EWARS remains unknown. An additional limitation was the challenge in assessing overall performance measures of the system, such as completeness, acceptability, and flexibility. We evaluated EWARS with regard to its objectives, but we could not extrapolate the effects of the system on the overall outbreak dynamics.

In conclusion, the magnitude and duration of the 10th and largest Ebola outbreak in DRC, occurring in an active conflict zone, highlighted the need for prompt, functional, and effective infectious disease surveillance systems. We have demonstrated that the EWARS implemented was a cost-effective component of this surveillance system. Our findings underscore the importance of early-warning systems, along with the necessity of ensuring efficiency and sustainability beyond the duration of the emergency response phase. As such, Integrated Disease Surveillance and Response is a relevant framework to further strengthen the International Health Regulations (2005) core capacities (*27**,**28*). The need to evaluate and learn from field implementation of surveillance systems in infectious disease outbreaks, even in such difficult contexts, is an opportunity to better understand response efforts and improve future responses (*29*).

Appendix Additional information about the evaluation of the Early Warning, Alert and Response System for Ebola virus disease, Democratic Republic of Congo, 2018–2020
