# Supplementary material for: Evaluation of Early Warning, Alert and Response System for Ebola Virus Disease, Democratic Republic of the Congo, 2018–2020
Source: Emerg Infect Dis. 2021 Dec;27(12):2988–98. doi: 10.3201/eid2712.210290 (PMC8632192; doi:10.3201/eid2712.210290)
Supplement: Appendix — Additional information about the evaluation of the Early Warning, Alert and Response System for Ebola virus disease, Democratic Republic of Congo, 2018–2020 [file 21-0290-Techapp-s1.pdf]

Article DOI: https://doi.org/10.3201/eid2712.210290

Evaluation of Early Warning, Alert and Response System for Ebola Virus Disease, Democratic Republic of the Congo, 2018–2020

Appendix

Appendix Table. Case definitions of Ebola virus disease for all ages as established by the World Health Organization

Suspected case	Confirmed case	Probable case	Non-case
Any person, alive or dead, suffering or having had a sudden onset of high fever and having had contact with:<br>-a suspected, probable or confirmed Ebola case;<br>-a dead or sick animal<br>OR: any person with sudden onset of high fever and >3 of the following symptoms: headaches; vomiting; anorexia/loss of appetite; diarrhea; lethargy; stomach pain; aching muscles or joints; difficulty swallowing; breathing difficulties; hiccup<br>OR: any person with inexplicable bleeding<br>OR: any sudden, inexplicable death.	Any suspected or probable cases with a positive laboratory result; laboratory-confirmed cases must test positive for the virus antigen, either by detection of virus RNA by RT-PCR, or by detection of IgM antibodies directed against Ebola.	Any suspected case evaluated by a clinician; OR any deceased suspected case (where it has not been possible to collect specimens for laboratory confirmation) having an epidemiologic link with a confirmed case.<br>Note: if laboratory specimens are collected in due time during the illness, the preceding categories are reclassified as "laboratory confirmed" cases and "non-case."	Any suspected or probable case with a negative laboratory result. "Non-case" showed no specific antibodies, RNA, or specific detectable antigens.

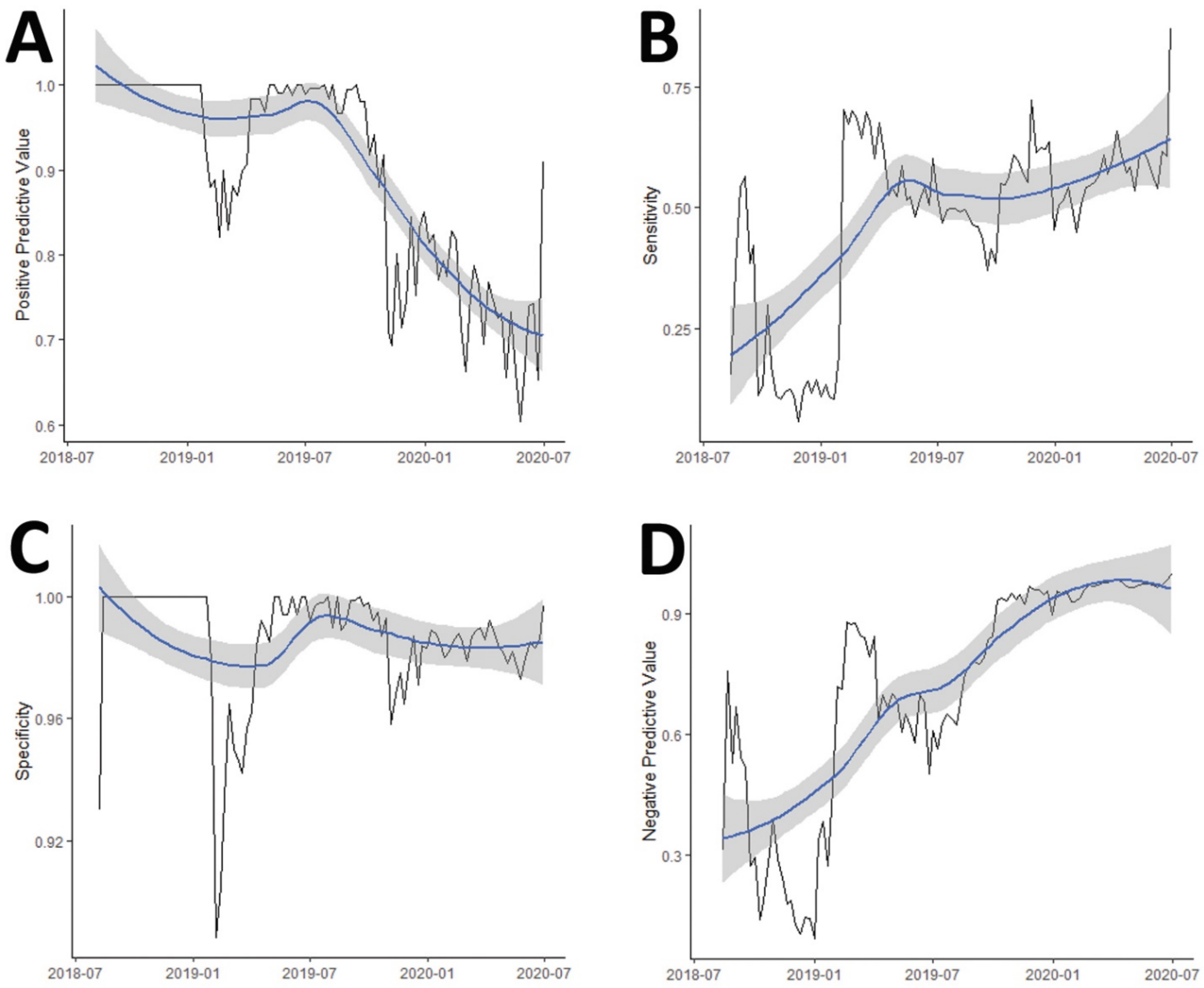

Appendix Figure. Weekly evolution of positive predictive value (A), sensitivity (B), specificity (C), and negative predictive value (D) of the Early Warning, Alert and Response System in Beni, Mutwanga, and Oicha, Democratic Republic of Congo, August 2018–June 2020.
